# Genomic similarity between gastroesophageal junction and esophageal Barrett's adenocarcinomas

**DOI:** 10.18632/oncotarget.10253

**Published:** 2016-06-23

**Authors:** Daysha Ferrer-Torres, Derek J. Nancarrow, Rork Kuick, Dafydd G. Thomas, Ernest Nadal, Jules Lin, Andrew C. Chang, Rishindra M. Reddy, Mark B. Orringer, Jeremy M. G. Taylor, Thomas D. Wang, David G. Beer

**Affiliations:** ^1^ Cancer Biology, Program in Biomedical Science, University of Michigan Medical School, Ann Arbor, Michigan, USA; ^2^ Section of Thoracic Surgery, Department of Surgery, University of Michigan Medical School, Ann Arbor, Michigan, USA; ^3^ Center for Cancer Biostatistics, Department of Biostatistics, School of Public Health, Ann Arbor, Michigan, USA; ^4^ Department of Pathology and Internal Medicine, University of Michigan, Ann Arbor, Michigan, USA; ^5^ Medical Oncology Department, Catalan Institute of Oncology, Barcelona, Spain; ^6^ Department of Medicine and Department of Biomedical Engineering, University of Michigan Medical School, Ann Arbor, Michigan, USA

**Keywords:** esophageal adenocarcinoma, gastroesophageal junction adenocarcinomas, molecular biomarkers, early detection

## Abstract

The current high mortality rate of esophageal adenocarcinoma (EAC) reflects frequent presentation at an advanced stage. Recent efforts utilizing fluorescent peptides have identified overexpressed cell surface targets for endoscopic detection of early stage Barrett's-derived EAC. Unfortunately, 30% of EAC patients present with gastroesophageal junction adenocarcinomas (GEJAC) and lack premalignant Barrett's metaplasia, limiting this early detection strategy. We compared mRNA profiles from 52 EACs (tubular EAC; tEAC) collected above the gastroesophageal junction with 70 GEJACs, 8 normal esophageal and 5 normal gastric mucosa samples. We also analyzed our previously published whole-exome sequencing data in a large cohort of these tumors. Principal component analysis, hierarchical clustering and survival-based analyses demonstrated that GEJAC and tEAC were highly similar, with only modest differences in expression and mutation profiles. The combined expression cohort allowed identification of 49 genes coding cell surface targets overexpressed in both GEJAC and tEAC. We confirmed that three of these candidates (CDH11, ICAM1 and CLDN3) were overexpressed in tumors when compared to normal esophagus, normal gastric and non-dysplastic Barrett's, and localized to the surface of tumor cells. Molecular profiling of tEAC and GEJAC tumors indicated extensive similarity and related molecular processes. Identified genes that encode cell surface proteins overexpressed in both Barrett's-derived EAC and those that arise without Barrett's metaplasia will allow simultaneous detection strategies.

## INTRODUCTION

Over 17,000 new cases of esophageal cancer will be diagnosed in the US in 2015, of which 61.5% will be esophageal adenocarcinomas (EAC) [1]. Over the past three decades the incidence of EAC in the US has risen at a rate of 7.5% per year [2], with other Western countries reporting similar increases [3, 4]. Currently this disease presents within a characteristic demographic, such that approximately 80% of new cases arise within Caucasian males over the age of 40 years [5, 6], and while the reasons for the rapid incidence increase are undetermined, it is clear that obesity, smoking and particularly chronic gastroesophageal reflux each play an important role. Advances in diagnostic and treatment approaches have improved short-term treatment responses, yet only one in five patients survive 5 years post-diagnosis [7]. The greatest obstacles to improved patient survival include an advanced stage at diagnosis and an incomplete response to chemoradiotherapy [8–10]. Evidence from several small-scale programs suggests an early diagnosis via adequate surveillance can dramatically improve EAC patient survival [11, 12], as well as reduce the need for aggressive chemoradiation [8]. Therefore there is a pressing need to implement efficient, accurate surveillance programs among high-risk populations.

We [13] and others [14, 15] are developing fluorescently-labeled imaging agents to enhance endoscopic detection of specific cell surface markers as a means to improve early stage EAC diagnosis. EAC arises from the precursor lesion, Barrett's esophagus (BE), which becomes dysplastic in a small minority of cases [16]. The presence of BE is currently the key factor for enrollment in existing surveillance programs, with histological evidence of dysplastic progression used as a trigger for treatment interventions, including surgical or endoscopic mucosal resection of high-grade dysplasia (HGD). However, 25-30% of EAC cases present with no histological evidence or history of BE [17, 18]. AC of the distal esophagus and junctional cardia do share many characteristics [reviewed by Carr *et al.* 19] and are currently treated using similar surgical and chemotherapy treatment strategies. At the University of Michigan Hospital, 30% of EACs arise at the GEJ that are not associated with the presence of BE [9]. Others have reported similar findings suggesting that GEJAC tumors make up a high proportion of EAC cases with no history of BE [17]. Here we applied molecular profiling technologies to assess the relationship between GEJAC and EAC, and expression profiling as an initial screen to identify potential cell surface markers for the detection of both GEJAC and EAC regardless of the prior presence of Barrett's mucosa.

## RESULTS

### GEJAC and tEAC demographics

Table 1 summarize the key characteristics of the 122 EAC tissues used for expression array analysis. There were no differences associated with gender, BMI, stage, node status, adjuvant treatment or tobacco usage between GEJAC and tEAC (Table 1), yet GEJAC cases presented at a slightly older mean age. We saw minimal differences in semi-quantitative measures for tumor histological characteristics, including desmoplastic response, differentiation or the degree of lymphocytic invasion within each tumor.

**Table 1:** Clinical characteristics

		GEJAC and tEAC			GEJAC no BE and tEAC with BE		
		GEJAC n=70 (100%)	tEAC n=52 (100%)	p-value	GEJAC no BE n=54 (100%)	tEAC with BE n=28 (100%)	p-value
Age	**median**	70.3	68.1	0.266^#^	70	69.7	0.914^#^
	**under 70**	26 (37.1%)	29 (55.8%)		20 (37.0%)	14 (50.0%)	
	**over 70**	44 (62.9%)	23 (44.2%)	0.0453^^^	34 (63.0%)	14 (50.0%)	0.345^^^
Gender							
	**male**	54 (77.1%)	47 (90.4%)		40 (74.1%)	25 (89.3%)	
	**female**	16 (22.9%)	5 (9.6%)	0.088^^^	14 (25.9%)	3 (10.7%)	0.152^^^
Weight category							
**under weight**	**BMI < 18.5**	1 (1.5%)	1 (2.3%)		1 (2.0%)	1 (4.0%)	
**normal weight**	**18.5 – 24.9**	23 (34.8%)	11 (25.0%)		17 (34.0%)	6 (24.0%)	
**over weight**	**25.0 – 29.9**	22 (33.3%)	22 (50.0%)		18 (36.0%)	12 (48.0%)	
**obese**	**30.0 and over**	20 (30.3%)	10 (22.7%)	0.962^@^	14 (28.0%)	6 (24.0%)	0.921^@^
Tumor stage							
	**I**	9 (12.9%)	4 (7.8%)		6 (11.1%)	3 (10.7%)	
	**II**	19 (27.1%)	10 (19.6%)		12 (22.2%)	8 (28.6%)	
	**III**	37 (52.9%)	30 (58.8%)		32 (59.3%)	14 (50.0%)	
	**IV**	5 (7.1%)	7 (13.7%)	0.11^@^	4 (7.4%)	3 (10.7%)	1.0^@^
Node status							
	**negative**	21 (30.0%)	8 (18.2%)		14 (25.9%)	7 (29.2%)	
	**positive**	49 (70.0%)	36 (81.8%)	0.189^^^	40 (74.1%)	17 (70.8%)	0.787^^^
Differentiation*							
	**well**	16 (22.9%)	4 (7.7%)		12 (22.2%)	3 (10.7%)	
	**moderate**	22 (31.4%)	19 (36.5%)		17 (31.5%)	9 (32.1%)	
	**poor**	32 (45.7%)	29 (55.8%)	0.066^@^	25 (46.3%)	16 (57.1%)	0.229^@^
Desmoplasia							
	**low**	25 (35.7%)	14 (26.9%)		19 (35.2%)	7 (25.0%)	
	**moderate**	21 (30.0%)	17 (32.7%)		15 (27.8%)	11 (39.3%)	
	**high**	24 (34.3%)	21 (40.4%)	0.379^@^	20 (37.0%)	10 (35.7%)	0.676^@^
Lymphocytic infiltration							
	**low**	24 (34.3%)	12 (23.1%)		19 (35.2%)	7 (25.0%)	
	**moderate**	28 (40.0%)	16 (30.8%)		20 (37.0%)	9 (32.1%)	
	**high**	18 (25.7%)	24 (46.2%)	0.039^@^	15 (27.8%)	12 (42.9%)	0.197^@^
Adjuvant treatment							
	**no**	54 (77.1%)	34 (65.4%)		42 (77.8%)	19 (67.9%)	
	**yes**	16 (22.9%)	15 (28.8%)	0.398^^^	12 (22.2%)	7 (25.0%)	0.78^^^
Tobacco usage							
	**no**	19 (28.8%)	16 (34.0%)		15 (29.4%)	10 (40.0%)	
	**yes**	47 (71.2%)	31 (66.0%)	0.68^^^	36 (70.6%)	15 (60.0%)	0.438^^^
BE status							
	**no BE**	54 (77.1%)	24 (46.2%)		100%		
	**+ BE**	11 (15.7%)	28 (53.8%)	3.13E-05^^^		100%	
	**unknown**	5 (7.1%)	0 (0%)				

# t-test:

^ Fisher's exact test:

@ Mantel-Haenszel Chi-square test:

* Seven tumors had pathologic evidence of signet ring cells (4 GEJACs and 3 tEACs)

We used pathology records to assess the presence or absence of BE. We found that 77% (54/70) of GEJACs arose in the absence of BE, significantly more frequent (p=3.13e-05) than among tEAC samples (46%; 24/52). Given the strength of this result, the consistency with previous studies [8, 10, 17, 20], and difficulties associated with clear demarcations on the basis of anatomical site of origin in advanced tumors, we chose to also compare the 54 GEJACs without evidence of underlying BE (perhaps representing GEJAC arising from cardia) to the 28 EACs with histologically confirmed BE. As shown in Table 1, when comparing these more rigorous subsets there were no differences in clinical characteristics. We also compared GEJAC with and without BE, and tEAC with and without BE across clinical parameters and found that for both tumor subsets the presence of BE was associated with a higher frequency of early stage tumors, as has been published previously [21]. Note that with only 11 cases of GEJAC with evidence of BE, therefore the GEJAC comparison is underpowered (Supplementary Table S1).

### Mutation comparisons of GEJAC and tEAC

**Figure 1:**
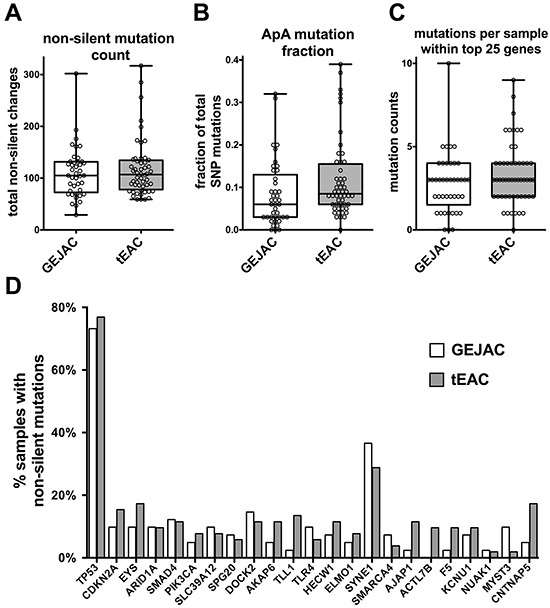
Mutation profiling comparison of GEJAC and tEAC Whole exome sequencing data were downloaded for a cohort of 149 normal-tumor pairs, with mutation type and frequency determinations performed as in Dulak *et al.* 2013 [22]. When looking at **A.** the total number of non-silent mutations in tEAC vs GEJAC we found no significance difference based on tumor type. There was a modest difference when **B.** only mutations with the ApA dinucleotide profile were considered with the Wilcoxon rank-sum test. When only the originally identified 26 significantly genes were considered there was **C.** no difference in the summated number per sample (p=0.134 by Wilcoxon rank-sum test), however **D.** there was significance when the collective mutation profiles for these genes were compared between GEJAC and tEAC by paired T-test.

Using whole-exome sequencing data from 149 normal and tumor pairs, samples with available pathology information assigning tumors as either tEAC (n=53) or GEJAC (n=41) in the original paper were chosen [22]. Figure 1A shows no significant differences in the total number of non-silent, protein-coding mutations between GEJAC and tEAC tumor groups, while Figure 1B shows that GEJAC mutations are significantly (p=0.02 Wilcoxon Rank-sum test) less likely to involve the ApA dinucleotide, a signature mutation associated with EAC [22, 23]. Although the incidence of all mutations shown in Figure 1 were previously described by Dulak and coworkers [22], specifically comparing incidence of mutations between GEJAC and tEAC was not presented in that study. Here we show that profiles of certain mutations (identified by Dulak) only slightly differ between GEJAC and tEACs. Among the 26 significantly mutated genes we found no difference in the overall mutation frequency (Figure 1C; p=0.13 Wilcoxon Rank-sum test) between GEJAC and tEAC, though the mutation profile was significantly different (p=0.047 by paired T-test; Figure 1D). While the most mutated gene in EAC by WES, *TP53* [22, 23], had a similar mutation frequency in both GEJAC and tEAC (75 and 77% respectively), several less frequently mutated genes (<15% of the cohort) showed a noticeably higher mutation rate in tEAC (*AKAP6*, *TLL1*, *AJAP1*, *ACTL7B*, *F5* and *CNTNAP5*) relative to GEJAC (Figure 1D), but the per gene mutation counts (ranging from 1 to 9 in either GEJAC or tEAC groups) were too small for individual gene statistical comparisons. Of the top 26 genes, only *MYST3* showed a notably higher mutation rate in GEJAC (9.8%; 4/41) compared to tEAC (<2%; 1/53).

We then considered GEJACs without BE vs tEACs with BE and saw the above results recapitulated, with a significantly lower fraction of ApA mutations in GEJAC without BE (p=0.023 by Wilcoxon Rank-sum test) and a significant difference in the distribution of mutations across the same 26 genes (p=0.04 by paired T-test), as well as similar individual gene profiles to those of the parent dataset listed above (Supplementary Figure S1).

### Unsupervised clustering of 122 tumors

**Figure 2:**
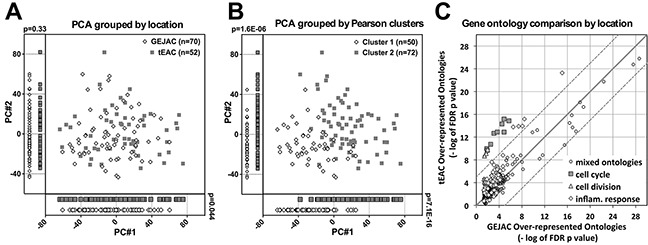
mRNA profiling comparison of GEJAC and tEAC All annotated probe sets (n=26,613), were standardized by subtracting the tumor cohort mean and dividing by the SD. The first two principal components (each with variance >5%: Supplementary Figure S3) were plotted and individual samples were assigned either **A.** a location (GEJ or tubular esophagus) or **B.** an unsupervised clustering assignment based on Pearson-correlation on the same 26,613 probe sets (Supplementary Figure S4) Visual and statistical comparison demonstrates minor expression differences between GEJAC and tEAC compared to class assignment by gene expression. **C.** Gene ontologies significantly over-represented (DAVID generated Benjamini adjusted p values <0.05) in GEJAC comparison to both the normal tissue groups were plotted against their tEAC equivalent using a – log 10 (p value) format. Dotted and continuous lines represent 10^5^ fold and 1:1 ratio markers respectively Results demonstrate that genes related to the cell cycle and broad inflammation ontology categories were more enriched in tEAC relative to normal tissues, compared to GEJAC.

We used PCA and unsupervised hierarchical clustering to investigate whether GEJAC represents a distinct, overlapping or indistinguishable subset of EAC, based on whole-genome expression profiling. For PCA we used all 26,613 annotated array elements across 135 mRNA samples (NE=8, NG=5, GEJAC=70, tEAC=52) and found that both types of normal samples were clearly separated from the tumors within the first 3 principal components (PC) (Supplementary Figure S2). To improve resolution within the cancer group we repeated PCA using only the 122 tumor samples (Figure 2). We then overlaid tumor location information, either GEJ or tubular esophagus, (Figure 2A), and assessed membership across PC1 and PC2, which each accounted for >5% of the total variance (Supplementary Figure S3). We performed unsupervised hierarchical clustering by Pearson correlation and complete linkage across all 135 mRNA profiles that resulted in 4 basic clusters; NE and NG groups, as well as two cancer clusters, designated C1 and C2 in Supplementary Figure S4. We then used membership in these two cancer clusters as an overlay for PCA and considered the same two PCs in order to provide a point of comparison (Figure 2B). We used the Wilcoxon rank sum test to assess whether there was a difference in sample distribution when location (Figure 2A) or unsupervised hierarchical clustering (Figure 2B) were used to group tumors. While the GEJAC and tEAC comparison did give a significant different across the first PC (p=0.044) we saw no obvious subgroups or division of samples. By contrast, and as expected, the difference resulting from the unsupervised hierarchical clustering of tumor samples by gene expression was visibly and significantly separated (p=7.1E-16), although still overlapping (Figure 2B). The results were very similar when only GEJAC without evidence of BE were compared to tEAC with BE using the same procedure outlined above (Supplementary Figure S5), demonstrating that the presence or absence of BE was not a key determinant.

### GEJAC and tEAC expression

Comparing the expression profiles of GEJAC and tEAC directly resulted in 1,368 differential probesets (ANOVA p-value < 0.01), although only 96 (7%) had a fold-change (FC) difference >1.5 (Supplementary Table S2). Given the low number of transcripts with meaningful FC shifts in this comparison, gene ontology analysis was conducted on all 1,368 using DAVID (1,183 unique Entrez gene IDs). This identified two over-represented gene categories (hsa05322: Systemic lupus erythematosus and hsa04514: Cell adhesion molecules), however only one gene, *HLA-DRB3*, (one of four genes common to both ontology categories) had >1.5-fold difference between GEJAC and tEAC (Supplementary Table S2).

As a more sensitive comparison, we identified genes that distinguished GEJAC and tEAC from normal (NG plus NE) tissue (ANOVA<0.01 and FC>1.5), then considered either increased (FC>1.5; Supplementary Table S3) or decreased (FC<0.67; Supplementary Table S4) in the cancer groups relative to normal tissues. As expected, ontology analyses on these lists for GEJAC and tEAC independently, revealed strong differences compared with normal tissues for both cancer groups, including cell cycle, immune response, extra cellular matrix structural factors, cell adhesion and digestion related categories; all previously reported in association with EAC. To compare the relative strengths of these ontology categories, we plotted the–log base 10 of Benjamini adjusted p values for ontologies over-represented within GEJAC against the corresponding values resulting in tEAC (Figure 2C). We considered >10^5^ fold difference between these matched p values (dotted lines marked on Figure 2C) to indicate a particular ontology category was more strongly represented within one cancer group. We assessed biologically relevant gene categories (relative to normal tissues) that might be more or less represented in GEJAC or tEAC. The majority of biological processes perturbed in EAC were similarly well represented in GEJAC and tEAC (Figure 2C), however cell cycle and inflammation-related categories were more strongly represented in tEAC relative to GEJAC (detailed in Supplementary Table S3).

### Transcripts associated with overall survival

By applying univariate COX analyses, we identified 1,289 Entrez genes (1,462 transcripts, including unknowns) with log-rank test p values <0.05 to overall survival in our treatment naïve cohort of 116 EACs from patients surviving more than 3 months post-surgery (Supplementary Table S5). This was very similar to the 1,331 transcripts (5% of 26,613) expected by chance. Of these just over half, 689 genes (784 transcripts), showed increased expression with increased risk (relative risk >1), which were overrepresented with members of the cadherin gene family residing in chromosomal band 5q31, in addition to a broad group of transcription-related genes (Supplementary Table S6). The contrasting set of 601 genes (679 transcripts), where reduced expression was associated with decreased overall survival, were over-represented by structural mitochondrial genes including a subset directly related to cellular respiration (Supplementary Table S7).

While no individual genes passed the false discovery adjusted significance threshold of 0.05, one gene, *ZNF217* had an FDR adjusted p=0.054 and a 2.3 hazard ratio (95% confidence interval of 1.6 to 3.3). The next strongest scores were for a cluster of 12 loci with FDR adjusted p values ranging from 0.29-0.3. These genes were associated with modest relative risk contributions of less than 2.5-fold with the majority showing increased expression and increased risk (Supplementary Table S8). Among these 13 genes the highest risk ratio was 5, for the pseudogene *GTF2IP1*, and the lowest was 0.4 to PIGW (Supplementary Table S8). Of interest were several zinc finger factors (*ZNF217*, *ZNF117*, *GTF2IP1* and *MEX3D*), though sparse *in silico* evidence links these genes.

**Figure 3:**
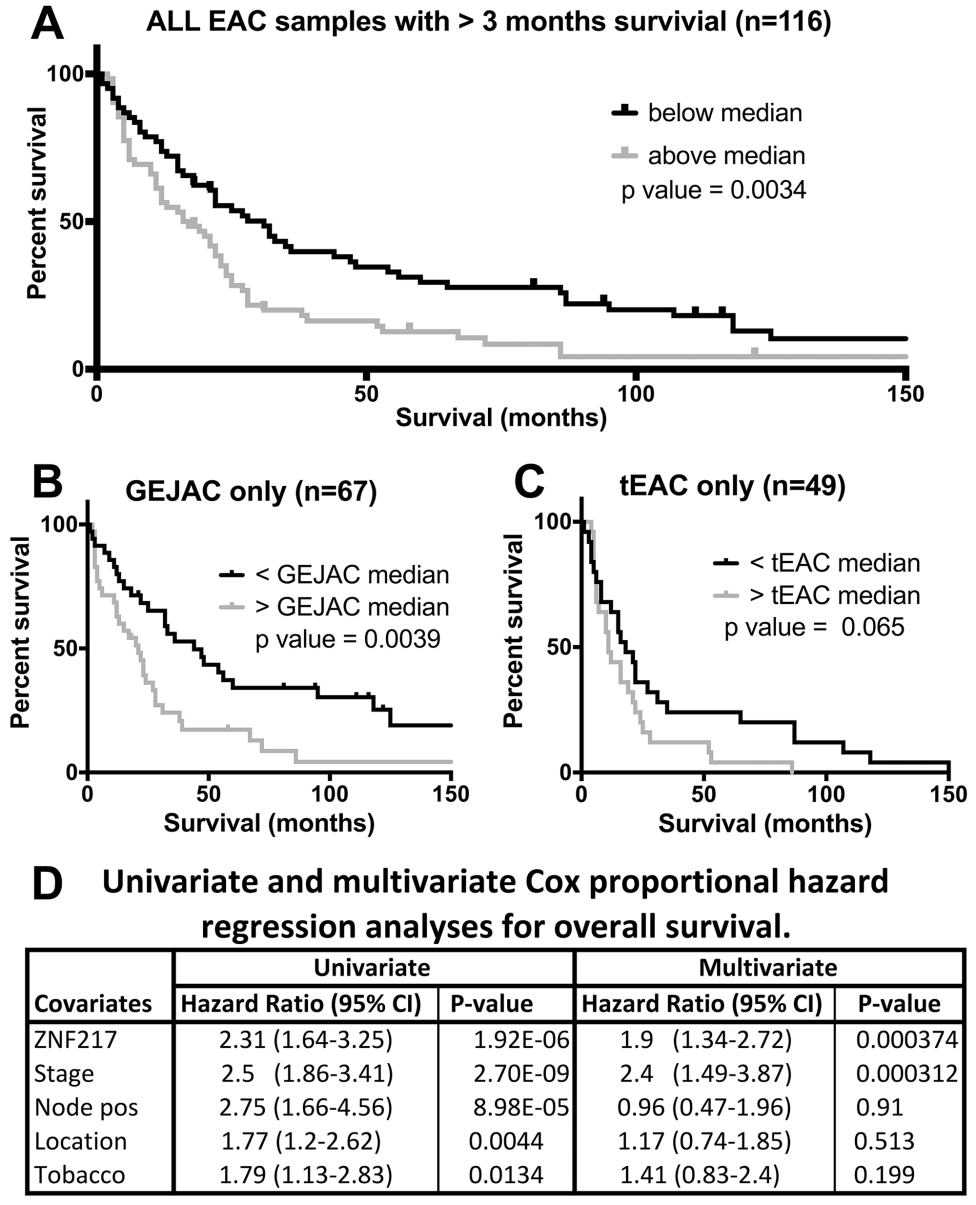
Univariate and multivariate analyses of *ZNF217* Univariate Kaplan-Meijer estimates with patients survived more than 3 months post-surgery stratified into high or low risk on the basis of median log2 normalized *ZNF217* expression. Plots demonstrate higher *ZNF217* expression as a risk factor whether **A.** all EACs, **B.** GEJAC only and **C.** tEAC only patients are considered. **D.** Shows tabulated comparisons of univariate and multivariate Cox proportional hazard components for *ZNF217* expression in conjunction with key clinical factors.

As the gene with the strongest correlation to survival in our cohort, we used Kaplan-Meijer plots to compare samples with high and low *ZNF217* expression for all 116 EACs, as well as GEJAC (n=67) and tEAC (n=49) subsets. We also assessed potential dependences on key clinical features using multivariate COX regression analysis (Figure 3A–3C). We used median expression across each tumor cohort to dichotomize high and low mRNA expression and reported log-rank p-value comparisons for each of these groups. These data confirmed a consistent, but modest survival benefit to EAC patients with low *ZNF217* expressing tumors (p=0.0034), with the same trend present in both GEJAC (p=0.0039) and tEAC (p=0.065) subsets.

We identified histological stage, node positivity, smoking history and tumor location as clinical variables with a univariate association to survival (Figure 3D). In the univariate analyses of tumor location (GEJAC vs tEAC) we saw that GEJAC was associated with a slight but significant improvement in overall survival by log-rank statistic (p=0.0044; Figure 3D and Supplementary Figure S6A). As can be seen in Table 1 and Supplementary Table S1, in our cohort there was a non-significant trend for GEJACs to present at an earlier stage, such that 40% of GEJACs presented with early stage disease (I or II) compared to 27.5% of tEACs. Siewert *et al.* [10, 24] reported similar findings, with GEJAC (AEG II) showed a shift towards earlier stage at presentation, however Clark *et al*. [17] and Curtis *et al.* [20] did not. When we restricted our comparison to early stage GEJAC vs early stage tEAC (Supplementary Figure S6B), or compare the late stage subsets (Supplementary Figure S6C), this relationship to overall survival disappeared (p=0.109 and p=0.169 respectively). We are unsure why, in our cohort, more GEJAC patients have an earlier presentation, however, given the strong correlation of disease stage to overall survival (Figure 3D) this difference in the distribution of disease stage may explain the improved survival for GEJAC patients. Multivariate analysis demonstrated that *ZNF217* over expression was independent of tumor stage, with other clinical variables having no significant impact on the model, including location (Figure 3D).

### Cell surface markers for GEJAC and EAC

We used a three-step procedure (Supplementary Figure S7) to identify overexpressed cell surface markers potentially useful for endoscope-based [13] detection of both GEJAC and tEAC. Firstly, differential genes distinguishing GEJAC (n=70) from NE and NG expression profiles were identified using both ANOVA (p<0.01) and fold-change (>2) thresholds, represented as Venn diagrams in Supplementary Figure S7: Step 1. This resulted in 396 transcripts for GEJAC and 534 when the same criteria were applied to tEAC (n=52) of which 359 were common to both lists (91% of the smaller list: Supplementary Figure S7: Step 2). We also used 2-fold rather than 1.5-fold to improve the prospect of qRT-PCR validation. Combined, the two lists totaled 571 transcripts, corresponding to 523 Entrez gene IDs. The broad gene ontology category GO:0005887 was used to identify plasma membrane associated factors within our list of genes overexpressed in GEJAC and/or tEAC and found 253 of the 523 encoded cell membrane proteins (Supplementary Figure S7: Step 3).

**Figure 4:**
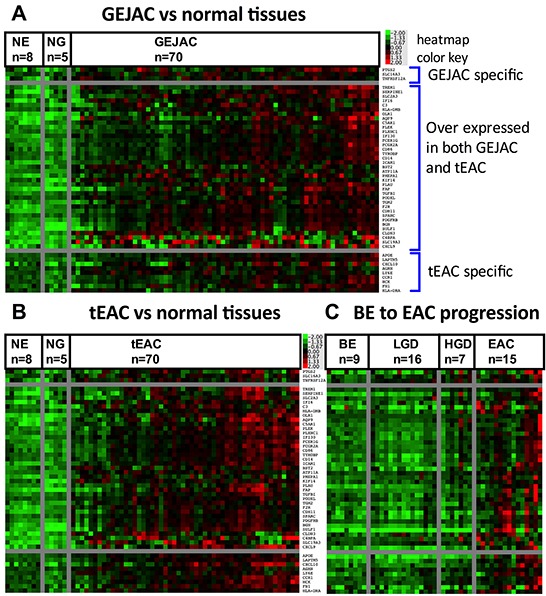
Heatmap of potential cell surface coding genes for GEJAC and tEAC ANOVA and fold-change based comparisons, in conjunction with Gene Ontology data, were used to identify 49 genes potentially over-represented in GEJAC and tEAC, as outlined in Supplementary Figure S1 and Methods. Mean normalized expression was then applied to these data to sort expression patterns across **A.** GEJAC and **B.** tEAC relative to mRNA from normal tissues, as well as **C.** EAC relative to BE samples ordered by histology, taken from Gene Expression Omnibus (GEO) Series ID GSE37203) (Silvers *et al.* [25]). In each figure plate the top three genes only passed the overexpression threshold in GEJAC, the lower nine only passed in tEAC, while the central listed genes were selected in both cancer types. While all genes are generally more highly expressed in tumor groups as compared to the represented non-cancerous tissues, there was considerable variation between tumor samples, with no clear pattern in relation to GEJAC and tEAC.

As a final step, we examined our prior BE-EAC progression cohort (GEO series GSE37203 [25]) that included Barrett's samples with no dysplasia (BE) (n=9), low-grade dysplasia (LGD) (n=15), high-grade dysplasia (HGD) (n=7) and EAC (n=15). We identified 684 transcripts overexpressed in EACs compared to BE without dysplasia, which included 151 genes, represented in GO:0005887 (Supplementary Figure S7). We then compared this list of genes overexpressed in EAC relative to BE to the list generated above and overexpressed in EAC (GEJAC and tEAC) relative to normal tissues. We found 49 membrane-associated genes that overlapped. Heat maps of these 49 genes for GEJAC vs normal tissues, tEAC vs normal tissues and the GSE37203 progression series (Figure 4A, 4B and 4C respectively) demonstrated that while expression was collectively higher in each tumor set, relative to non-cancer tissues, each individual gene was high in only a subset of tumors. The 3 genes that passed selection thresholds for GEJAC, but not tEAC, (top genes in Figure 4A, 4B and 4C) were also overexpressed in a number of tEACs. Similarly, the last 9 genes listed in each Figure 4 panel passed our expression threshold in tEAC only, but were overexpressed in a similar portion of GEJACs. Thus the GEJAC and tEAC group-specific expression trends were very similar across the 49 genes.

While the mean expression for each tumor group represented in each Figure 4 panel was higher than the non-cancer sample groups for each of the 49 genes, each gene had a number of individual cancer samples with expression levels comparable to normal tissues. This lower expressing subset varied for each gene thus to discriminate the majority of EACs from surrounding tissues, multiple genes are required. While some degree of correlated expression was evident among the 49 genes, several had more unique expression profiles, including *CLDN3* and *SLC19A3*, potentially representing valuable additions to a detection panel.

Seventeen of the 49 potential cell surface marker genes identified were previously reported in association with EAC including *PLAU, PTGS2* and *SPARC* which showed increased EAC expression, relative to BE, in multiple studies (summarized in Supplementary Table S9). In addition, we recently showed TGM2 is overexpressed on the surface of EAC cells [26]. In the current study, we chose to validate *CDH11, ICAM1* and *CLDN3* as examples of potential cell surface markers common to subsets of both GEJAC and tEAC.

**Figure 5:**
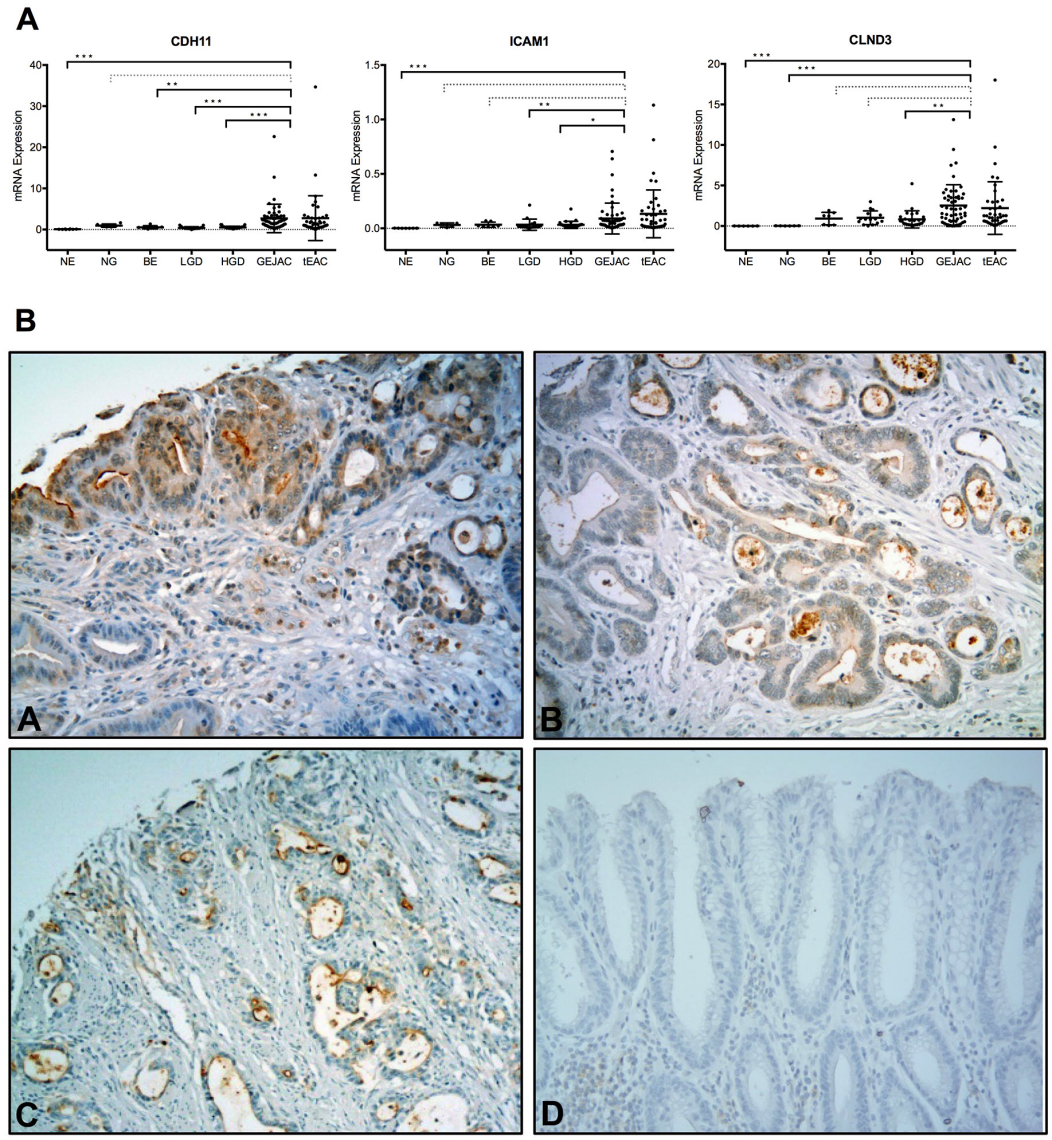
qRT-PCR and protein validation of potential cell surface markers Three genes, *CDH11*, *ICAM1* and *CLDN3,* were chosen to confirm mRNA overexpression in EAC tumors, and demonstrate tumor cell surface staining of gene products. **A.** Relative qRT-PCR expression levels were determined for an esophagus-related panel of cDNA samples using the ABI PRISM® 7900HT technology and *GAPDH* as a reference gene, as described in Methods. GEJAC and tEAC groups were not significantly different for any gene (p=0.12, 0.65 and 0.17 by WMU). The combined cancers were compared to NE, NG, BE and HGD groups (grey no significant comparison, * p<0.05, ** p<0.01, *** p<0.001 by MWU) for each of the 3 genes. **B.** We then used a TMA containing histologically-confirmed EAC tissues to demonstrate that commercially available antibodies for A. CDH11, B. CLDN3, **C.** ICAM1 and **D.** no primary antibody negative control stained cell surface profiles localized to HGD and tumor cells.

Using an expanded cohort including available arrayed samples (7 NE, 5 NG, 58 GEJAC, 46 EAC) together with additional samples (1 NE, 1 NG, 7 BE, 19 LGD and 29 HGD), qRT-PCR was used to confirm overexpression of selected candidate genes in cancer relative to normal and precancerous tissues (Figure 5). Supplementary Figure S8 demonstrates that Pearson-correlation analyses *CDH11, ICAM1* and *CLDN3* among Human Gene 2.1 ST arrayed samples indicate consistent correlations between log2 array and relative expression (qRT-PCR) data (rho values of 0.84, 0.81 and 0.89 respectively). While each gene showed a clear difference between NE and either GEJAC or tEAC, differences were less distinct among non-cancer columnar tissues (NG and the BE groups: BE, LGD, HGD) (Figure 5A) suggesting that these genes would only be useful for distinguishing cancer foci from pre-cancer and normal tissues, rather than markers for identifying high-risk epithelium. Using our TMA with commercially available antibodies as shown in Figure 5B, cell surface expression of these markers was observed in HGD and EACs, although high-level expression was only observed in small subset of tumors. For 14 EACs we had matching mRNA and TMA data. Although this overlapping subset was small, there was a trend towards specimens with higher mRNA levels staining strongly for the corresponding protein (data not shown). While protein detection sensitivity may be an issue, there are many biological considerations that influence protein to message ratios, such that mRNA levels can only be considered as a screening tool to identify likely candidates for protein validation.

## DISCUSSION

We found that GEJACs have significantly less histological evidence of BE when compared to tEAC, yet molecular comparisons of these tumor classifications using DNA mutation and mRNA expression profiling suggest only minor differences, even when the presence or absence of BE was taken as a co-discriminator. Minor differences were also observed in both mutation profiles, with less ApA mutations among GEJACs (a recognized characteristic of the EAC mutation profile [22, 23]), and over-represented gene ontologies, with less cell cycle and immune response factors overexpressed in GEJAC. Together these observations may suggest that a subset of EACs arise as a result of a more extreme set of conditions requiring more prominent mucosal defense and an increasing the likelihood of initiating the formation of BE (with goblet cells), although resulting tumors arise via the same set of mutagenic triggers.

Although pathology confirmed >70% viable tumor in each cancer specimen is it possible that associated normal tissue present may have masked GEJAC and tEAC cellular differences. In this case microdissection, rather than macrodisection may better discriminate GEJAC and tEAC. However, it should be noted that were true, then our mutation analysis would still have detected the differences between tumor cell types. We believe the subtle differences we observed by both expression and mutation analyses suggest that GEJAC and tEAC cancer cells are similar. As we move into IHC screening of our cell surface markers, we will be able to discern not only whether these proteins localize to the cell surface, but also which cells are staining. Markers that highlight stromal cells, rather than tumor cells will not be prioritized for validation as the relationship between stroma, activated stroma and tumor cells is still an emerging field of investigation. Expression data revealed 1368 transcripts were significantly different between GEJAC and EAC, and more than expected by chance (266), but only 96 transcripts (7%) demonstrated >1.5-fold difference between tumor groups. As expected from these minor differences, PCA analysis showed that tumor location (GEJAC or tEAC) was not a strong influence on gene expression profiling.

These results are consistent with epidemiological studies that demonstrate most risk factors for GEJAC and tEAC are shared, with subtle patient differences in obesity-related factors, reflux and gender [reviewed by 19, 27]. A significantly reduced association between BE histology and adenocarcinomas arising at the GEJ has often been observed [8–10, 17, 20], as we confirm here. The difference in BE rate does not translate into molecular differences suggests that the founding cell type(s), and pathway(s) for GEJAC and tEAC are shared. Molecular investigations of EAC indicate a heterogeneous disorder with different combinations of changes leading to cancer, suggesting the existence of molecular subtypes, as is the case for other common cancers. Unsupervised clustering of expression profiles in Figure 2 demonstrated that the underlying molecular characteristics were much stronger than minor differences attributed to GEJAC vs tEAC. Perhaps the underlying tumor causation spectrum is influenced by tumor location, though the specific investigation of this hypothesis is beyond the scope of the current study. When examining the DNA copy number variations of 27 GEJAC tumors, Isinger-Ekstrand *et al*. [28] also found that junctional AC profiles mirrored those of tEAC and were distinct from changes frequent in non-cardia gastric cancers. We report that both expression profiling and mutation analyses suggest a shared etiology for GEJAC and EAC of the distal esophagus. This holds true whether group distinction was based solely on tumor location, or whether the absence of BE was included as a co-discriminator. It should also be noted that a lack of evidence of BE at the time of surgery does not exclude the possibility that it was either missed (present in esophageal sections other than those reviewed for histology) or that it was overrun by the cancer leaving no evidence at surgery.

The known association of EAC with BE has led to surveillance biopsy protocols with the intent to detect early cancer in these patients. The reduced incidence of BE, however, suggests that a large proportion of individuals at risk for GEJAC are unlikely to be considered for routine screening. The development of novel fluorescently-labeled peptides for endoscopic identification of early cancer in the esophagus [13] has increased the potential for the detection of early Barrett's-associated adenocarcinomas, with the promise of improving patient outcomes. The strong similarity between Barrett's-associated EAC and GEJAC, as shown in the present study, suggests that potentially useful peptides could be developed that would identify cancers of both the lower esophagus and GEJ, regardless of the presence of Barrett's esophagus.

Univariate COX analysis for overall survival against all 26,613 annotated transcripts showed that over expression of *ZNF217* mRNA represented the strongest gene-based risk within our cohort, with both GEJAC and tEAC samples showing support for this association (Figure 3). While *ZNF217* was the strongest, and just short of FDR-adjusted significance, several other genes show evidence of an association, though as with *ZNF217*, their relative risk contributions were small (<2.5 fold: Supplementary Table S8). In other cancer types, both mRNA and protein levels for ZNF217 have been shown to correlate with patient outcomes, including breast, ovarian, colon and prostate cancer types [recently showed by 29, and reviewed by 30, 31]. These associations generally correlate with the presence of gain of chromosome 20q, a frequent event in EAC [32–35]. Both ZNF217 protein and mRNA tumor expression have been associated with 20q13 copy number for several tumor types [36–38]. Geppert *et al*. [39] used FISH to demonstrate that the presence of chromosomal gain involving *ZNF217* predicted stage-independent survival in 130 EAC patients. While based on copy number, these data are consistent with our mRNA findings.

Several lines of evidence implicate ZNF217 as a key player in the regulation of the epithelial to mesenchymal transition (EMT), including the discovery of *CDH1* as a direct repression target [40] and that *ZNF217* expression can be directly regulated by several EMT-related miRNAs, including miR-24 [41], miR-203 [42] and miR200c [43]. In prostate cancer miR-203 exists in a double negative feedback loop with the EMT transcription factor *SNAI2*, along with *ZNF217* [44] and miR-203 was previously shown to differentiate EAC from BE and decreased expression associated with poorer EAC patient outcome [45, 46].

Using ChIP-seq analysis Frietze *et al.* [47] showed that ZNF217 associates with the repressive histone marks H3K27ac and H3K4me1. Transgenic models with up regulation of *ZNF217* expression stimulate mesenchymal transition through the activation of Snail1 and Twist [29]. Thus epigenetic remodeling, with ZNF217 as a key component, could be a central feature in explaining the dynamic nature of EMT [48].

Our expression profiling analysis has revealed 49 genes encoding potential cell surface markers that demonstrate transcriptional overexpression in EAC tumors compared to normal and pre-cancerous tissue. We confirmed tumor-specific overexpression for three genes (*CDH11, ICAM1* and *CLDN3)* using qRT-PCR, and demonstrated protein localization specific to the cell surface of tumor cells by IHC. In addition, we have recently demonstrated that TGM2 was also overexpressed and present on the cell surface of EAC cells [26], while both *PTGS2* (COX2) and *TNFRSF12A* are known to increase during the transition from BE to EAC [49, 50]. The products of several genes from our potential cell surface list are suspected of playing key roles in more general cancer-related activities such as immunosuppression/evasion (*CD14* and *CD86*), cell migration (*ICAM*, *CDH11*) and proliferation (*TGFB1*, *PMEPA1*, *PDGFRL*, *SLC19A3*). Other markers on this list may have confounding issues, for example *OLR1* has shown strong squamous cell staining at the leading edge of the epithelial surface while *SLC2A3* (GLUT3) expression is known be elevated in the tissue of smokers [51]. These factors, along with protein expression gradient, and overexpression frequency will need to be considered in the construction of a specific panel of markers to aid in the identification of early cancers. Ultimately we aim to apply a multiplexed panel of peptides using multispectral scanning fiber endoscope technology [52] to improve the success of histology-based screening programs for early EAC detection.

## MATERIALS AND METHODS

### Sample cohort

All samples were obtained following written, informed patient consent according to the approval and guidelines of the University of Michigan institutional review board. Tissues were obtained from patients undergoing esophagectomy for adenocarcinoma within the University of Michigan Health System between 1991 and 2012, without preoperative radiation or chemotherapy. A portion of each specimen was immediately frozen in liquid nitrogen and stored at −80°C until use. All resected cancers underwent pathological analysis, and only those indicating adenocarcinomas arising either within 1 cm above and 2 cm below the GEJ (Siewert type II [10]) or within the distal (tubular) esophagus, more than 1 cm above the GEJ (tEAC), were included in this study. A certified pathologist (DGT) performed categorical or semi-quantitative histopathological assessment of the sections as follows; tissue type (squamous, BE, cardia, gastric), tumor type (AC), differentiation (well, moderate, poor), desmoplastic response (weak, moderate, high) and inflammatory response (weak, moderate, high). We noted histological evidence of signet ring cells in seven tumors (4 GEJAC and 3 tEAC). Cryostat sectioning was used to select regions containing >70% tumor cellularity prior to DNA or RNA isolation. Height and weight data, at the time of surgery, were extracted from patient records and used to determine BMI category as follows: ‘underweight’ (BMI < 18.5), ‘normal’ (BMI 18.5 to 24.9), ‘overweight’ (BMI 25 to 29.9) and ‘obese’ (BMI 30.0 and above). Adjuvant treatment was considered positive when a standard chemo and/or radio treatment commenced within not more than three months after primary resection. Pathology reports were also reviewed regarding the presence of BE. A sample was considered positive for BE when the pathologist noted goblet cells among the columnar tissue at the margin of tumor sections, or when BE was noted in the resected material. Using this information, we have included additional analyses based on comparing the subset of GEJACs with no evidence of BE to the subset of tEACs where the presence of BE was noted as described below.

### Whole exome sequencing comparison of GEJAC and tEAC

WES data generated by Dulak *et al.* [22] was used to investigate differential mutation profiling within GEJAC and tEAC subgroups with variant calling, annotation and sample characteristics provided in the original publication. We compared all non-silent mutations observed in GEJAC (n=41) or tEAC (n=52) samples using the Wilcoxon Rank-sum test, as well as a paired Student T-test (two-sided) comparisons of the non-silent mutations within the 26 genes significantly mutated within the entire WES cohort (n=149) as originally identified [22] using the MutSig algorithm [53]. We also conducted analyses in which we compared the subset of GEJAC samples where histology did not note BE (n=35; 85% of the GEJAC mutation cohort) to the subset of tEAC samples where BE was noted (n=42; 81% of the tEAC mutation cohort).

### mRNA profiling

Total RNA was purified from normal esophageal squamous (NE; n=8), normal gastric (NG; n=5) epithelium and adenocarcinomas arising at both the gastro-esophageal junction (GEJAC; n=70), and within the ‘tubular’ esophagus (tEAC; n=52) using miRNeasy spin columns (Qiagen, Valencia, CA), including on-column DNAse I incubation, according to the manufacturer's instructions. RNA samples with RIN scores greater than 6.0 (Bioanalyzer; Agilent Technologies, Palo Alto, CA), were submitted to the University of Michigan Cancer Center Genomics Core for cDNA synthesis, cRNA amplification (Ambion WT Expression Kit; Life Technologies, Grand Island, NY) and hybridization to Human Gene ST 2.1 arrays (Affymetrix, Santa Clara, CA) according to the manufacturer instructions. Expression values for each gene were estimated using the robust multi-array average (RMA) method [54] in the Bioconductor package [55] and log2-transformed. Analyses were restricted to the 26,613 coding and non-coding genes for which annotation details were available, including HUGO Gene Nomenclature Committee (HGNC) approved gene symbol and Entrez Gene ID.

### Principal component analysis and unsupervised clustering

We used Cluster (version 3.0) to perform Principal Component Analysis (PCA) of expression array data to visualize the relationship between sample groups. Mean normalized, batch adjusted log 2 expression data for 26,613 annotated array elements were applied to PCA, either using all 135 samples (8 NE, 5 NG, 70 GEJAC and 52 tEACs) or just the 122 tumor samples. To generate two-dimensional plots, we compared the top principal components (PC), ranked by eigenvalues, which individually explained the highest levels of the total variance, using 5% as a minimum threshold for investigation. Among these components those that best demonstrated the separation between sample groups were graphed. Typically, this meant the top two PCs were compared.

The software packages Cluster (version 3.0) and Treeview (Java version 3.0 [56]) were used to generate and graph unsupervised hierarchical clustering of the 135 expression profiles using Pearson correlation with average linkage using 26,613 annotated array elements. Data were normalized to tumor means for each gene to aid in dendrogram visualization. This analysis resulted in normal samples clustering together and tumors separating into two groups, with mixed GEJAC and tEAC membership in each of these clusters (Supplementary Figure S4). Given that GEJAC and tEAC groups were not distinct by either PCA or hierarchical clustering, the Pearson correlation cluster membership was overlaid onto the PCA graphs, as a comparison to demonstrate how well the sample cohort could be separated. We used this as a comparison purely to more clearly demonstrate that PCA incompletely discriminated between GEJAC and tEAC.

### Gene ontology analysis of expression array data

The arrays were run in two batches, the first batch holding 8 NE, 5 NG, and 35 GEJAC, while the second batch consisted of 52 tEAC and an additional 35 GEJAC. We adjusted for batch effects by adding probe-set specific constants to each value in the second batch such that the probe-set means for GEJAC's in batch 2 agreed with those of batch 1. When fitting a one-way analysis of variance (ANOVA) model with means for each of the four tissue types, we reduce the degrees of freedom in the mean-squared-error and F-tests by 1 to account for this batch adjustment. Mean group expression ratios (typically >1.5 or 2-fold increase/decrease), in combination with an ANOVA test of p<0.01, were used to select differentially expressed genes between groups. Enrichment testing for over-represented gene ontology terms was performed using the DAVID website with the appropriate platform-specific background gene list (“HuEx-1_0-st-v2”) and default algorithm settings [57, 58]. Individual ontology categories with false discovery adjusted (Benjamini) p values <0.05 were reported, though we applied the modular enrichment analysis (MEA) based Functional Annotation Clustering feature built into DAVID to assess redundant gene categories and to group similar gene sets under appropriate descriptors [59]. Both batch-normalized and raw expression data for this experiment were deposited into the Gene Expression Omnibus (GEO series GSE74553).

### Identification of genes associated with overall survival

Of the 122 tumors used in this study, there were 2 patients who died from surgical complications within a month of surgery, and a further 4 patients who died within 3 months of surgery (3 GEJAC and 3 tEACs combined). In order to reduce the possibility of surgical complications confounding survival data, we chose to use the identified 116 patients who survived more than 3 months following surgery. For these patients the average survival time was 38.7 months (range: 3 to 251 months), and an average follow-up time of 94.2 months (range: 18 to 242 months) for surviving participants. Using univariate analyses, we determined that of the available clinical variables stage, node status, tumor location and smoking status each showed an association to overall survival (Figure 3D). We applied univariate COX analysis for all 26,613 annotated transcripts and applied FDR adjustment to the resulting log-rank (Mantel-Cox) test p-values. We considered genes with an FDR adjusted p value <0.05 to provide a significant association to overall survival. Survival associations were plotted (Kaplan-Meijer) using dichotomized mRNA expression, with cohort median expression as a cutoff. Multivariate analyses were used to assess whether the survival associations for significant genes were independent of stage, node status, tumor location and smoking status.

### Identification of GEJAC/tEAC expressing genes for cell surface proteins

To identify cell surface-coding genes selectively overexpressed in both GEJAC and tEAC, we applied a three-step procedure schematically represented in Supplementary Figure S7. In step 1, we asked that GEJAC vs NE and GEJAC vs NG comparisons both gave p<0.01 and a fold-change (FC) >2 and that this also was true for comparisons of tEAC vs NE and tEAC vs NG. In step 2 we selected the subset of genes indicated as being “plasma membrane” by Gene Ontology (GO:0005887), as listed within the COMPARTMENTS subcellular localization database, which resulted in 5 162 potentials among the 26,613 transcripts [60]. For step 3, we then analyzed the resulting list of genes in our previously published, independent data-set of 9 BE, 7 BE+LGD, 8 LGD, 7 HGD, 15 EAC assayed on Affymetrix U133A arrays (GEO Series GSE37203) in order to compare cancer (EAC) and non-dysplastic pre-cancer (BE) expression levels by ANOVA and fold-change (Supplementary Figure S7) [25].

### qRT-PCR validation

cDNA synthesis was performed using the High Performance RT-PCR Kit (Life Technologies, Grand Island, NY) according to the manufacturer's instructions. Total RNA from 58 GEJAC and 46 tumors from the ST 2.0 array, 53 BE (7 without dysplasia, 17 LGD and 29 HGD), 6 NG and 8 NE samples were available for real-time (qRT-PCR) validation of the selected gene transcripts. qRT-PCR reactions primers were designed using Primer-BLAST [61] (*CDH11*: 5′-GCACGAGACCTATCATGCCA-3′, 3′-CTGTCTGTGCTTCCACCGAA-5′, *ICAM1*: 5′-GTA TGAACTGAGCAATGTGCAAG-3′, 3′-GTTCCACCCG TTCTGGAGTC-5′, *CLDN3*: 5′- TCGGCCAACACCA TTATCCG-3′, 3′-GTACTTCTTCTCGCGTGGGG-5′, *ZN F217*: 5′- CTCCGGGCCACTTTACACTT-3′, 3′-TCTCT TTTGTGCCATGCTGTT-5′) or previously published [GAPDH: 62]. Annealing temperatures were determined and optimized using Cepheid SmartCycler (Cepheid, Sunnyvale, CA). Samples were run using the ABI PRISM® 7900HT Sequence Detection System according to the manufacturer's instructions and analyzed using relative quantitation utilizing GAPDH as the reference gene. Technical validation was assessed by correlating (Pearson rho) log2 of relative qRT-PCR expression values with matched log2 ST 2.1 array data for each validation gene (Supplementary Figure S8). GAPDH was chosen because it was highly expressed (mean log2 expression of 7.57 across all samples) with a minimal mean difference between normal and tumor samples (1.03-fold for 13 normal vs 122 tumor samples) within our ST 2.1 array data and is known to be an effective reference for esophageal samples [63].

### Immunohistochemistry and tissue microarray (TMA)

A TMA was constructed as described by Kononen *et al* [64] containing 122 cores derived from the resected tissue from 73 EAC patients, including 60 tEAC, 3 GEJAC, 22 BE, 9 metastatic lymph nodes, and 14 normal tissues. Five μm sections were used for immunohistochemistry as previously described [25]. CDH11 (Cat# 32-1700, Life Technologies), ICAM1 (Cat# ab53013, Abcam, Cambridge, MA) and CLDN3 (Cat# 18-7340, Thermo Scientific, Pittsburgh, PA) monoclonal antibody were used at dilutions of 1:500, 1:250 and 1:100, respectively, after microwave citric acid epitope retrieval for 20 minutes and lightly counterstaining with hematoxylin. Each sample was then scored 0-3 corresponding to absent, light, moderate, or intense staining.

## SUPPLEMENTARY FIGURES AND TABLES



















## Abbreviations

GEJ: gastroesophageal junction
GEJAC: gastroesophageal junctional adenocarcinoma
tEAC: esophageal adenocarcinoma originating within the tubular esophagus, above the GEJ
EAC: esophageal denocarcinoma, including both GEJAC and tEAC
PCA: Principal Component Analysis
