# Supplementary material for: Genomic similarity between gastroesophageal junction and esophageal Barrett's adenocarcinomas
Source: Oncotarget. 2016 Jun 23;7(34):54867–82. doi: 10.18632/oncotarget.10253 (PMC5342387; doi:10.18632/oncotarget.10253)
Supplement: Supplementary file 1 [file oncotarget-07-54867-s001.pdf]

Genomic similarity between gastroesophageal junction and esophageal barrett's adenocarcinomas

SUPPLEMENTARY FIGURES AND TABLES

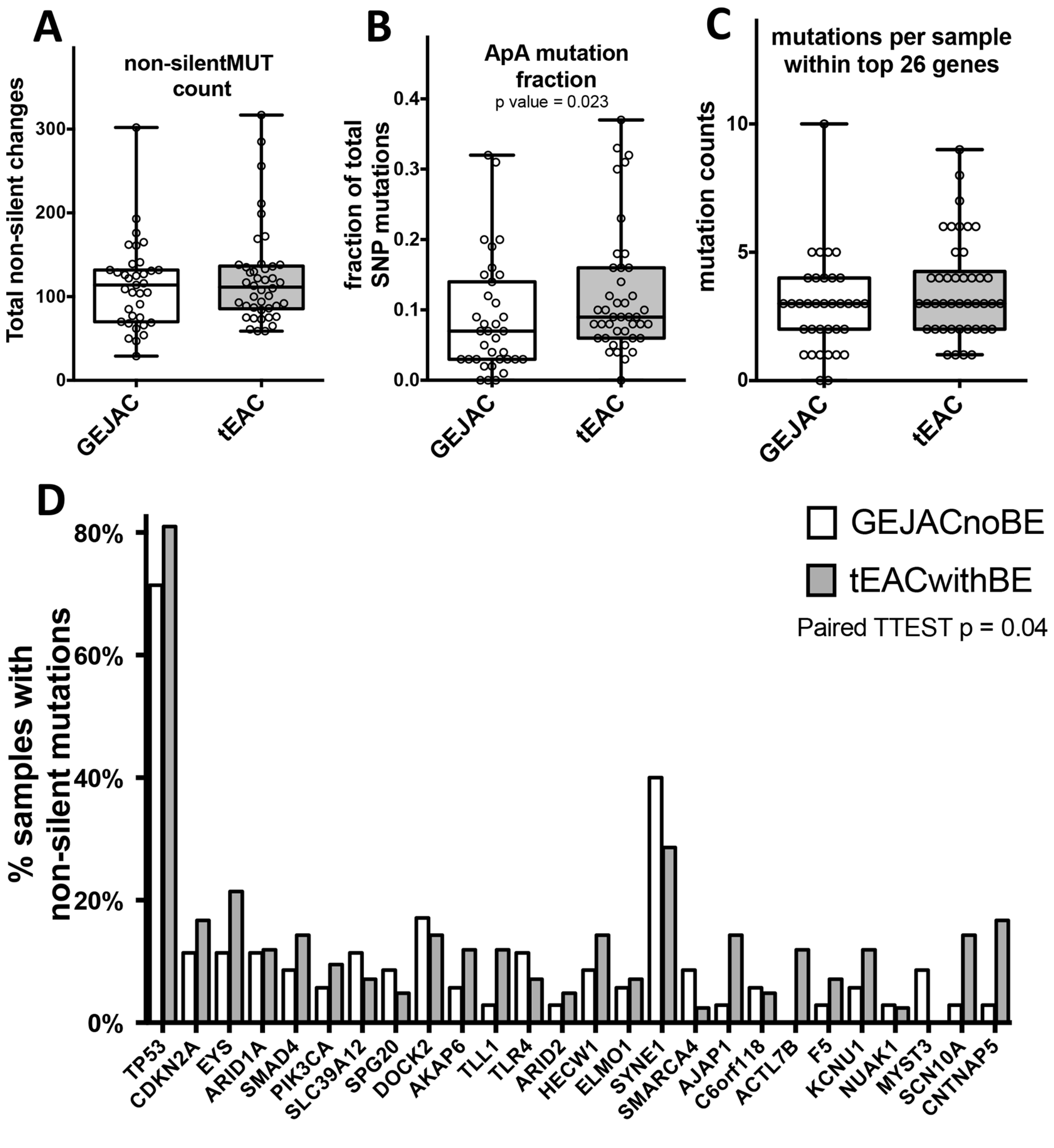

Supplementary Figure S1: Mutation profiling comparison of GEJAC without BE and tEAC with BE. Whole exome sequencing data for GEJAC samples without BE histology (GEJACnoBE: n=35) and tEAC samples where BE histology was noted (tEACwithBE: n=42) were extracted from a cohort of 149 normal-tumor pairs, with mutation type and frequency determinations performed as in Dulak *et al.* 2013. When looking at **A.** the total number of non-silent mutations in tEACwithBE vs GEJACnoBE we found no significance differences. There was a modest difference when **B.** only mutations with the ApA dinucleotide profile were considered with the Wilcoxon rank-sum test. When only the originally identified 26 significantly mutated genes were considered there was **C.** no difference in the summated number per sample, however **D.** there was significance when the collective mutation profiles for these genes were compared between GEJACnoBE and tEACwithBE by paired T-test.

A

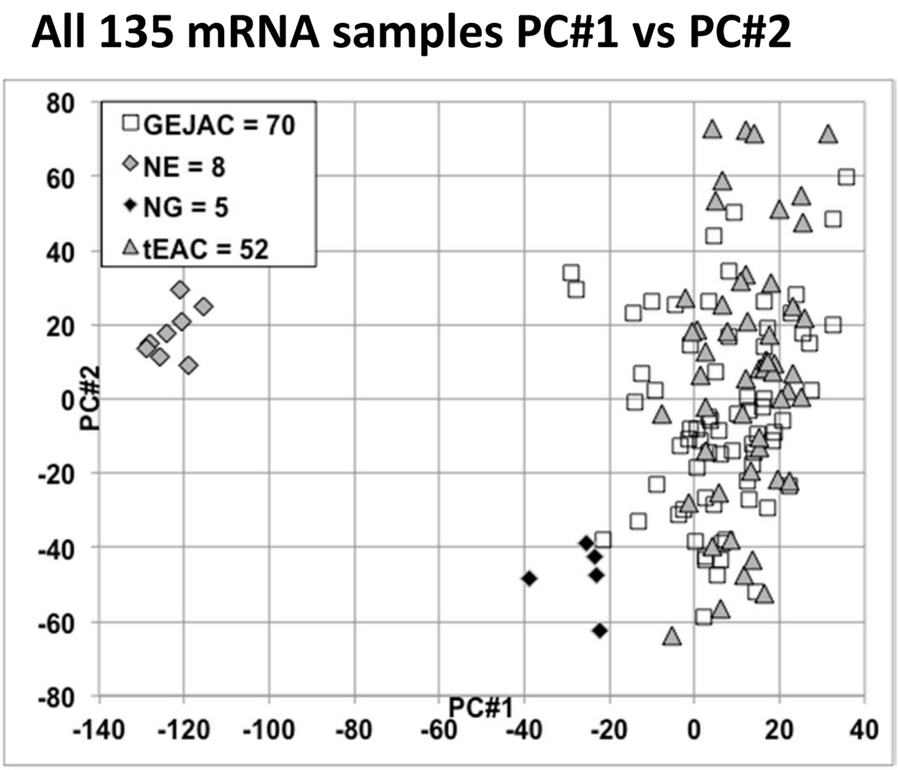

B

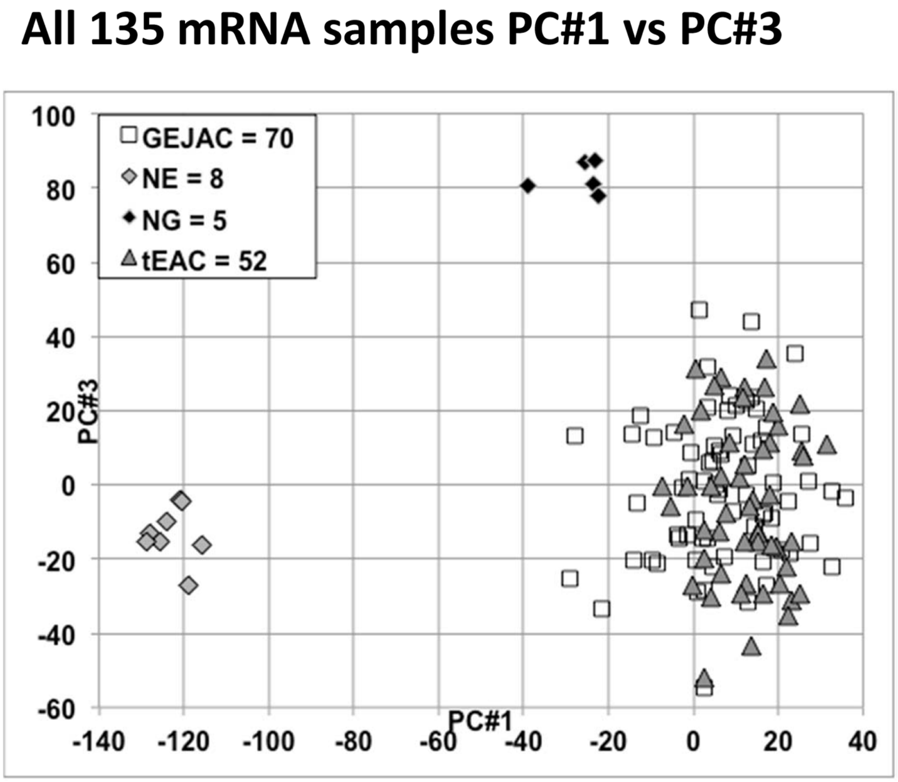

Supplementary Figure S2: PCA analysis in all 4 mRNA profiling groups. All annotated probe-sets (n=26,613) were standardized by subtracting the mean of all 135 samples and dividing by the SD. **A.** the first two and **B.** first and third principal components were plotted and individual samples were assigned to their four histological groupings to demonstrate clear separation of normal tissues (NE and NG) but no separation of tumor groups (GEJAC and tEAC).

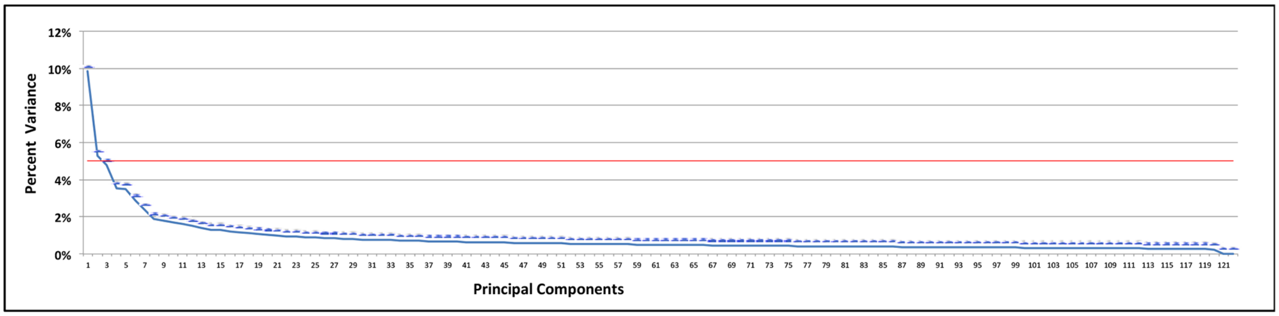

Supplementary Figure S3: The fraction of variance in each principle component of tumor only PCA analysis. All annotated probe-sets (n=26,613), were standardized by subtracting the 122 tumor sample mean and dividing by the SD. The percent of total variance attributed to each principle component were then plotted to show that the top two components (PC#1 and PC#2) each explain more than 5% of the total variance.

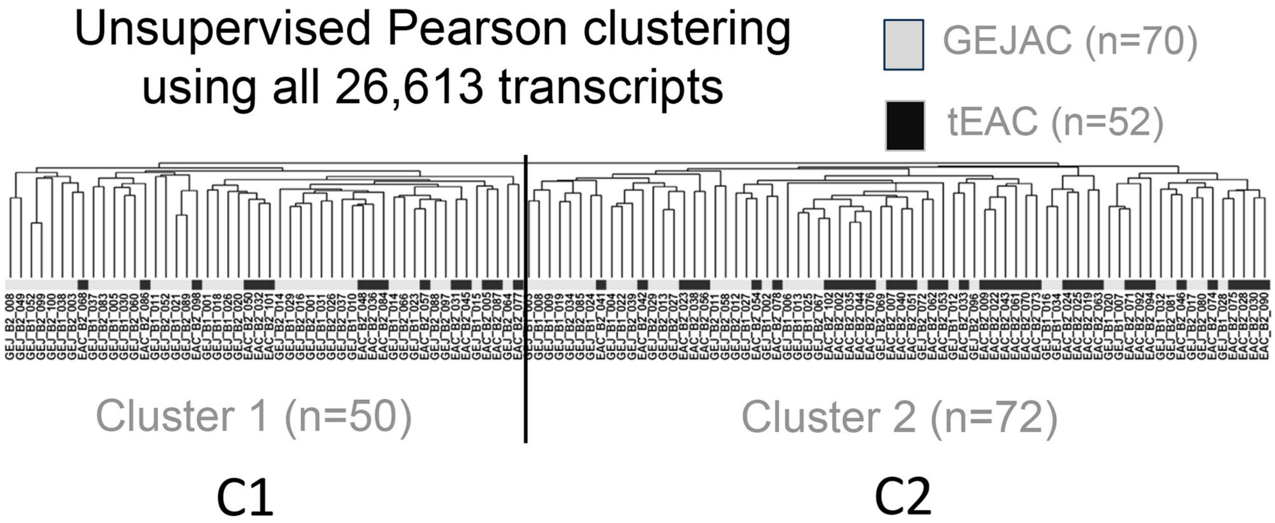

Supplementary Figure S4: Hierarchical clustering in 122 tumor mRNA samples. Unsupervised clustering assignment for 122 tumor samples based on Pearson-correlation and complete linkage on all 26,613 probe-sets.

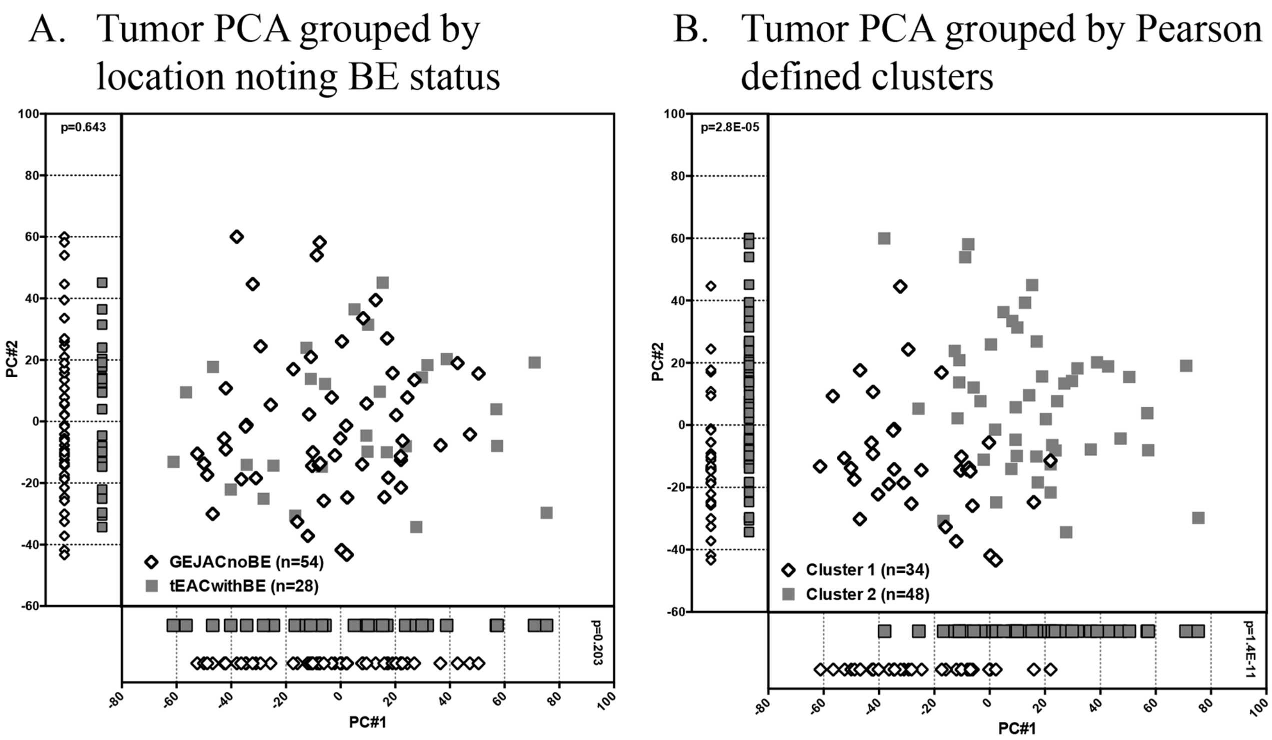

Supplementary Figure S5: PCA comparison of GEJACnoBE and tEACwithBE. All annotated probe-sets (n=26,613) were standardized by subtracting the tumor cohort mean and dividing by the SD. Only GEJACs with no histological evidence of BE (GEJACnoBE; n=54) and tEACs with BE (tEACwithBE; n=28) were considered. The first two principal components (each with variance >5%: (Supplementary Figure S4) were plotted and individual samples were assigned either **A.** a location (GEJ or tubular esophagus) or **B.** an unsupervised clustering assignment based on Pearson-correlation on the same 26,613 probe sets (Supplementary Figure S4). Visual and statistical comparison demonstrated minor expression differences between GEJACnoBE and tEACwithBE compared to class assignment by gene expression, as was seen when all GEJAC and tEAC samples were compared (Figure 2).

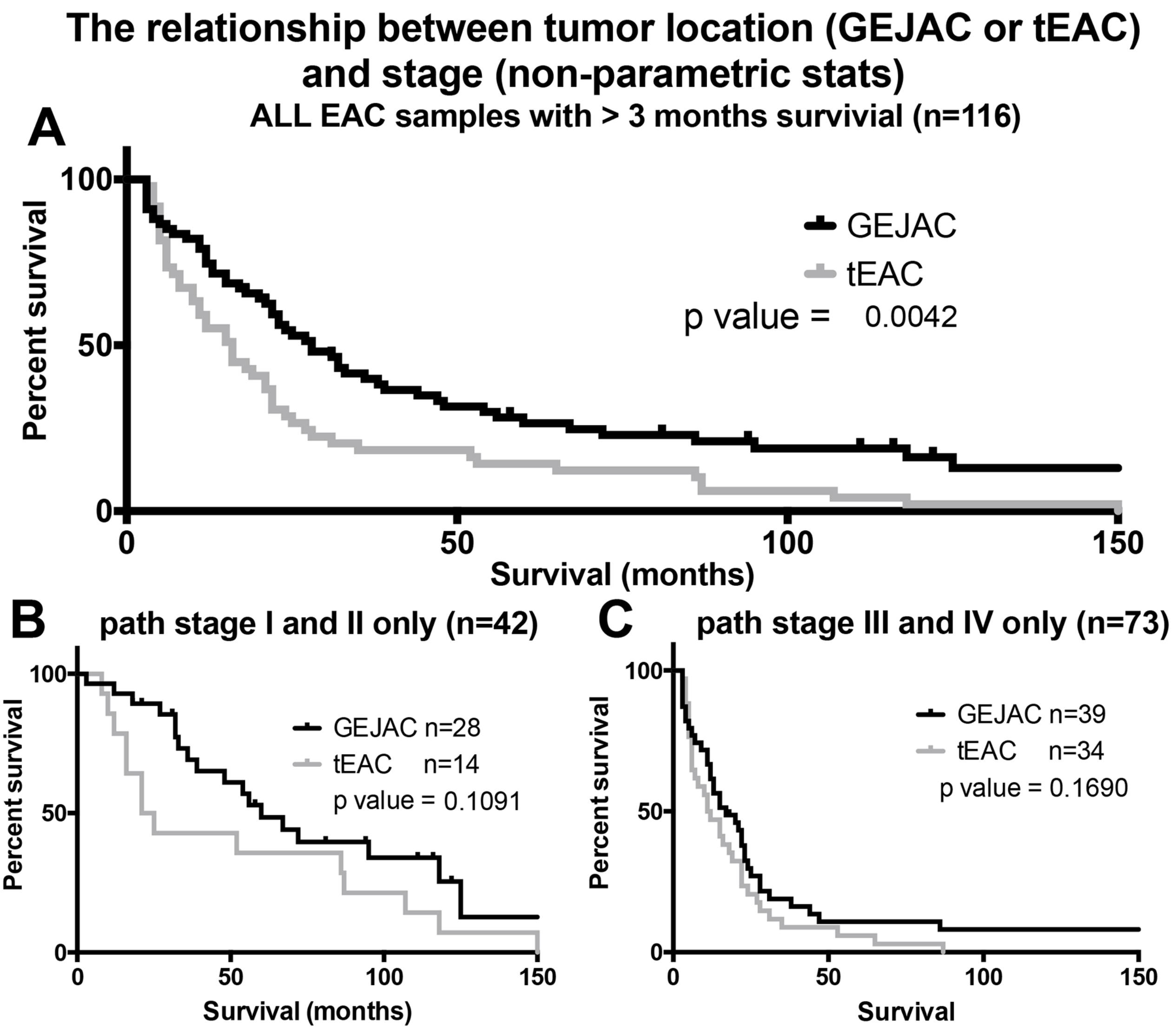

D Univariate and multivariate Cox proportional hazard regression analyses for overall survival (Location)

Covariates	Univariate		Multivariate	
	Hazard Ratio (95% CI)	P-value	Hazard Ratio (95% CI)	P-value
Location	1.77 (1.2-2.62)	0.0044	1.36 (0.862-2.14)	0.187
Stage	2.5 (1.86-3.41)	2.70E-09	2.2 (1.38-3.48)	0.000833
Node pos	2.75 (1.66-4.56)	8.98E-05	1.03 (0.50-2.12)	0.932
Tobacco	1.79 (1.13-2.83)	0.0134	1.64 (0.98-2.74)	0.0589

Supplementary Figure S6: Survival analyses comparing GEJAC and tEAC. A. Univariate survival analysis shows GEJAC had improved survival over tEAC, however by looking at **B.** early and **C.** late stage tumors separately, as well as **D.** multivariate analysis, indicated this association is dependent upon tumor stage. Kaplan-Meijer plots and log-rank p=values were generated in Prism while tabulate univariate and multivariate analyses were made using the coxph module in R.

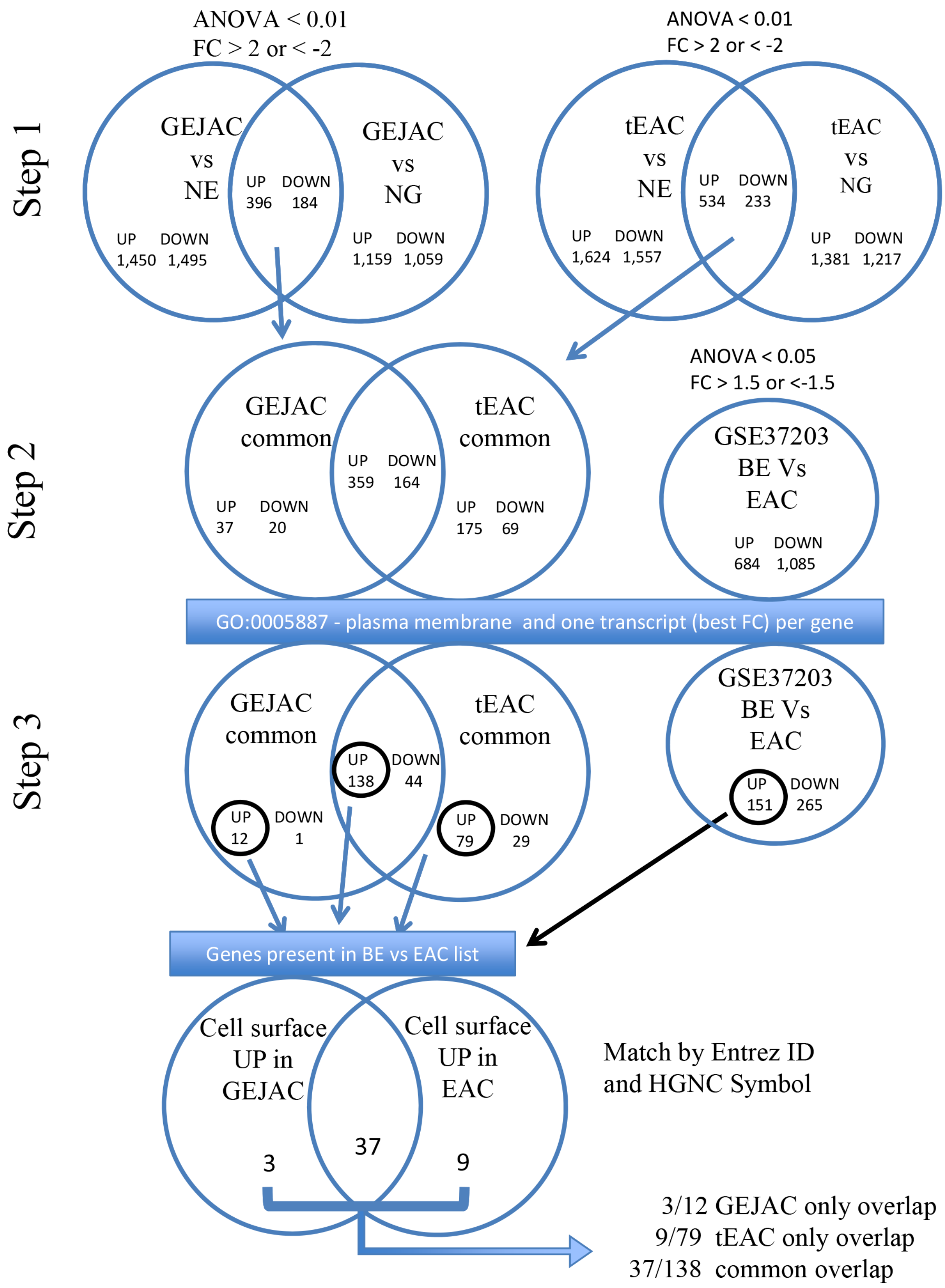

Supplementary Figure S7: Steps used to identify potential cell surface markers for GEJAC and tEAC using expression profiling data. Beginning with all 26,613 probe-sets the schematic demonstrates the bioinformatic steps as we combine comparisons between each normal tissue group (NE and NG) and each tumor group (GEJAC and tEAC). Genes in the combined subset with known plasma membrane associations by GO were then compared to those similarly upregulated in EAC relative to BE within our previously published progression cohort (GEO ID: GSE37203). The resulting 49 genes represent potential cell surface factors upregulated in EAC (both GEJAC and tEAC) relative to surrounding normal and pre-cancerous tissues. Numbers represent Entrez IDs (genes) on each list.

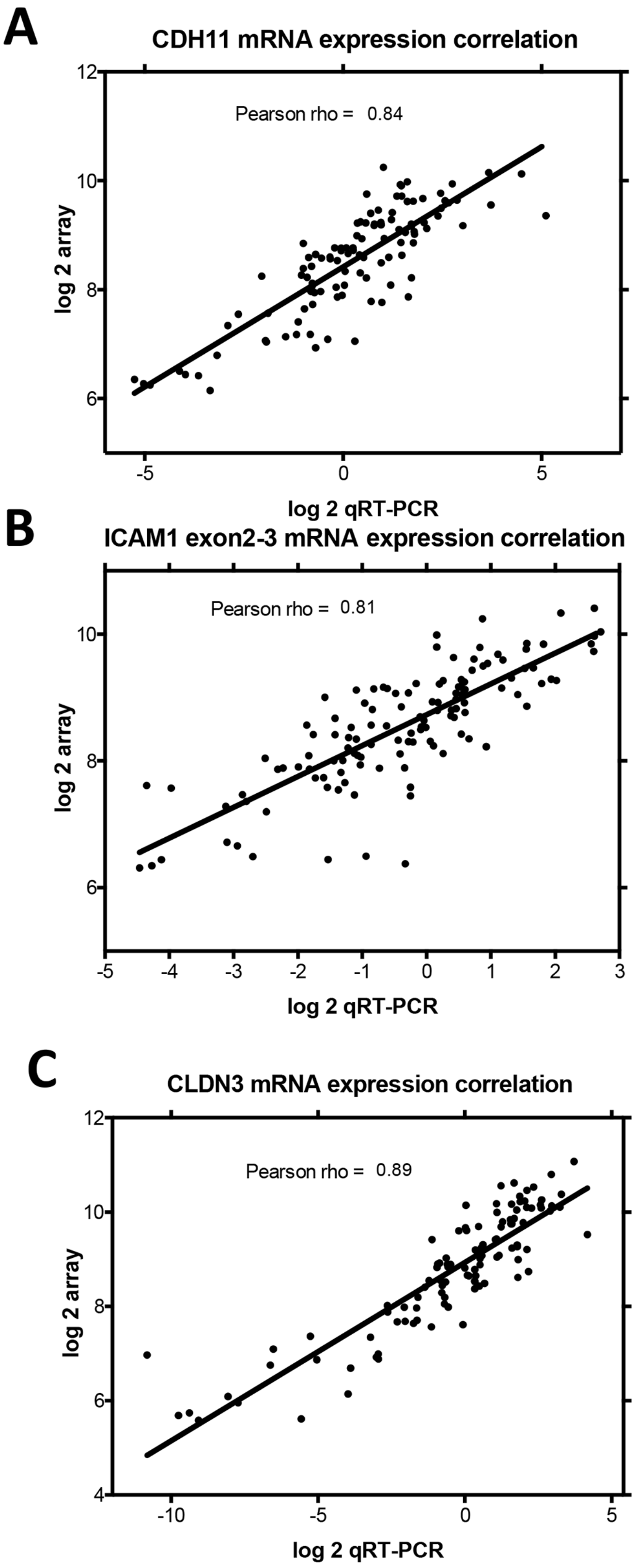

Supplementary Figure S8: Validation of ST 2.1 array data by qRT-PCR. Pearson-correlation analyses for **A.** *CDH11*, **B.** *ICAM1* and **C.** *CLDN3* comparing log2 normalized Human Gene 2.1 ST arrayed samples and relative expression (qRT-PCR relative to *GAPDH*) data.

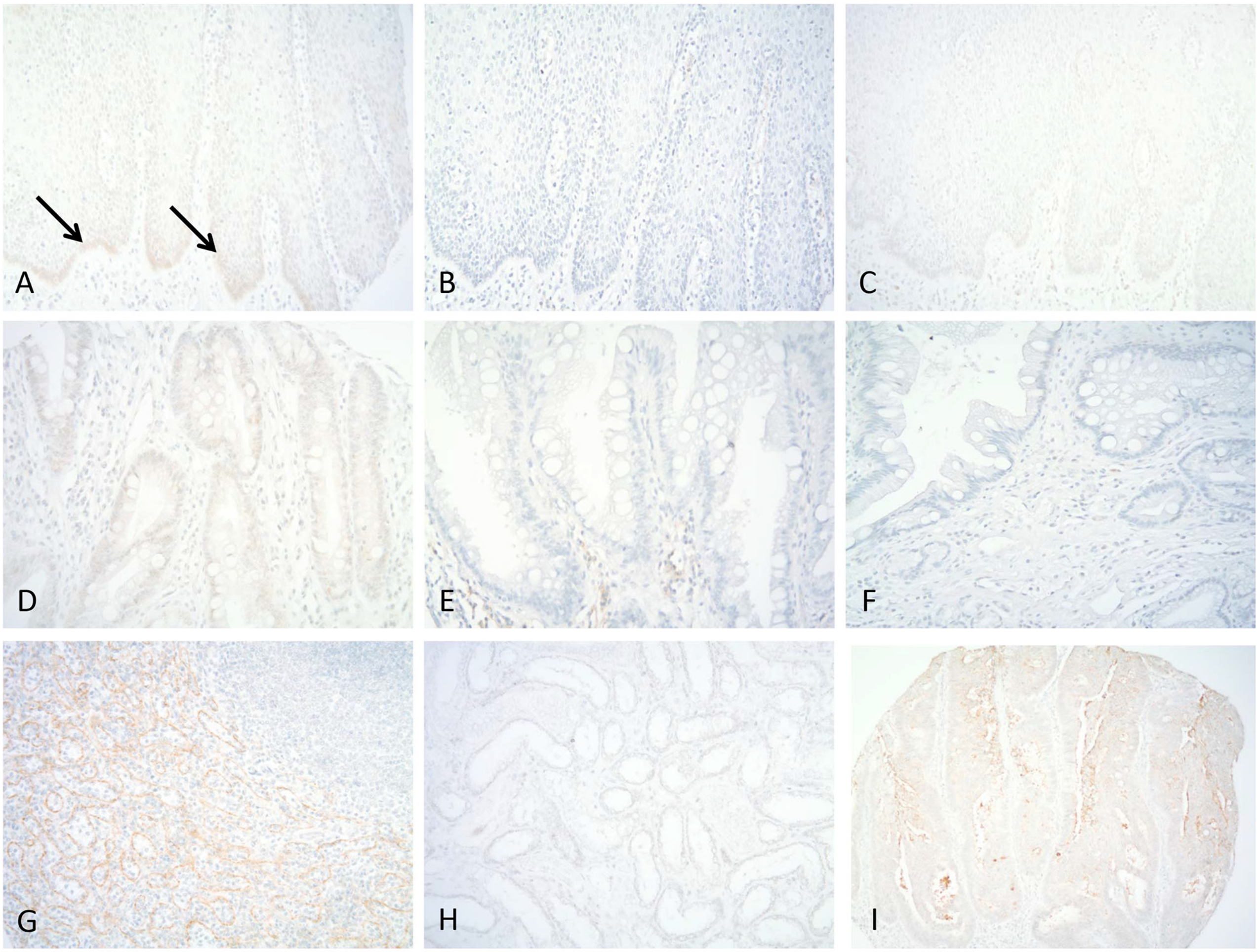

Supplementary Figure S9: Immunohistochemical analysis of CDH11 A, D, G., ICAM1 B, E, H. and Claudin3 C, F, I. using esophageal tissue microarrays. The normal esophageal squamous mucosa shows low level immunoreactivity to these three gene products A, B, C), with the exception of CDH11 that shows positive staining in the basal cells within the proliferative zone (arrows, A). Intestinal-type Barrett metaplasia is primarily negative for all three gene products although slight staining of the epithelium is detected with CDH11. Positive and negative controls included in the TMA showing normal spleen with areas having both positive and negative CDH11 staining (G). Normal kidney is negative for ICAM1 (H) and colon adenocarcinoma demonstrates strong cell surface Claudin3 immunoreactivity (I). Original magnifications A-C and G-I: 100X; D-F: 200X.

Supplementary Table S1: Clinical characteristics of BE related subtypes for each cancer group

		GEJAC with and without BE			tEAC with and without BE		
		**GEJAC no BE n=54* (100%)**	**GEJAC with BE n=11 (100%)**	**p-value**	**tEAC no BE n=24 (100%)**	**tEAC with BE n=28 (100%)**	**p-value**
Age	**median**	70	72.3	0.695[#]	66.2	69.7	0.173[#]
	**under 70**	20 (37.0%)	4 (36.4%)		15 (62.5%)	14 (50.0%)	
	**over 70**	34 (63.0%)	7 (63.6%)	1.00^	9 (37.5%)	14 (50.0%)	0.412^
Gender							
	**male**	40 (74.1%)	9 (81.8%)		22 (91.7%)	25 (89.3%)	
	**female**	14 (25.9%)	2 (18.2%)	0.718^	2 (8.3%)	3 (10.7%)	1.00^

(*Continued*)

		GEJAC with and without BE			tEAC with and without BE		
		GEJAC no BE n=54* (100%)	GEJAC with BE n=11 (100%)	p-value	tEAC no BE n=24 (100%)	tEAC with BE n=28 (100%)	p-value
Weight category							
**under weight**	**BMI < 18.5**	1 (2.0%)	0 (0.0%)		0 (0.0%)	1 (4.0%)	
**normal weight**	**18.5 – 24.9**	17 (34.0%)	4 (36.4%)		5 (26.3%)	6 (24.0%)	
**over weight**	**25.0 – 29.9**	18 (36.0%)	3 (27.3%)		10 (52.6%)	12 (48.0%)	
**obese**	**30.0 and over**	14 (28.0%)	4 (36.4%)	0.722@	4 (21.1%)	6 (24.0%)	0.906@
Tumor stage							
	**I**	6 (11.1%)	2 (18.2%)		1 (4.3%)	3 (10.7%)	
	**II**	12 (22.2%)	6 (54.5%)		2 (8.7%)	8 (28.6%)	
	**III**	32 (59.3%)	3 (27.3%)		16 (69.6%)	14 (50.0%)	
	**IV**	4 (7.4%)	0 (0.0%)	0.0398@	4 (17.4%)	3 (10.7%)	0.0747@
Node status							
	**negative**	14 (25.9%)	5 (45.5%)		1 (5.0%)	7 (29.2%)	
	**positive**	40 (74.1%)	6 (54.4%)	0.275^	19 (95.0%)	17 (70.8%)	0.0544^
Differentiation							
	**well**	12 (22.2%)	4 (36.4%)		1 (4.2%)	3 (10.7%)	
	**moderate**	17 (31.5%)	3 (27.3%)		10 (41.7%)	9 (32.1%)	
	**poor**	26 (46.3%)	4 (36.4%)	0.371@	13 (54.2%)	16 (57.1%)	0.841@
Desmoplasia							
	**low**	19 (35.2%)	5 (45.4%)		7 (29.2%)	7 (25.0%)	
	**moderate**	15 (27.8%)	6 (54.6%)		6 (25.0%)	11 (39.3%)	
	**high**	20 (37.0%)	0 (0.0%)	0.0837@	11 (45.8%)	10 (35.7%)	0.793@
Lymphocytic infiltration							
	**low**	19 (35.2%)	4 (36.4%)		5 (20.8%)	7 (25.0%)	
	**moderate**	20 (37.0%)	6 (54.6%)		7 (29.2%)	9 (32.1%)	
	**high**	15 (27.8%)	1 (9.1%)	0.437@	12 (50.0%)	12 (42.9%)	0.187@
Adjuvant treatment							
	**negative**	42 (77.8%)	8 (72.7%)		15 (62.5%)	19 (67.9%)	
	**positive**	12 (22.2%)	3 (27.3%)	0.706^	8 (33.3%)	7 (25.0%)	0.757^
Tobacco usage							
	**no**	15 (29.4%)	16 (34.0%)		15 (29.4%)	10 (40.0%)	
	**yes**	30 (70.6%)	31 (66.0%)	0.68^	36 (70.6%)	15 (60.0%)	0.438^
BE status							
	**no BE**	100%			100%		
	**+ BE**		100%			100%	
	**unknown**	0 (0.0%)	0 (0.0%)				

#, t-test: ^, Fisher's exact test: @, Mantel-Haenszel Chi-square test: *, 5 individuals with unknown BE status were removed

Supplementary Table S2: Transcripts differentially expressed between GEJAC and tEAC (ANOVA p < 0.01) - gene list and DAVID ontology results.

See Supplementary File 1

Supplementary Table S3: Genes up-regulated in cancer (GEJAC and tEAC) relative to normal tissues

See Supplementary File 2

Supplementary Table S4: Genes down-regulated in cancer (GEJAC and tEAC) relative to normal tissues

See Supplementary File 3

Supplementary Table S5: Genes with log-rank p-values less than 0.05 for univariate Cox analysis in 116 EACs from patients surviving > 3 months post-surgery

See Supplementary File 4

Supplementary Table S6: DAVID ontology results for anti-survival genes (log-rank p-values < 0.05 and Rel risk > 1) for univariate Cox analysis in 116 EACs from patients surviving > 3 months post-surgery

See Supplementary File 5

Supplementary Table S7: DAVID ontology results for pro-survival genes (log-rank p-values < 0.05 and Rel risk < 1) for univariate Cox analysis in 116 EACs from patients surviving >3 months post-surgery

See Supplementary File 6

Supplementary Table S8: Top overall survival genes from univariate Cox analysis

See Supplementary File 7

Supplementary Table S9: Subset of our 49 cell surface genes previously associated with EAC in multi-gene studies

See Supplementary File 8
